# Health-related quality of life disparities among vestibular schwannoma patients under different treatment regimens: A systematic review and meta-analysis

**DOI:** 10.1016/j.bas.2026.106074

**Published:** 2026-05-03

**Authors:** M. Rutenkröger, L. Brandes, L. Hänsel, M. Scheer

**Affiliations:** a Department of Medical Psychology, University Medical Center Hamburg-Eppendorf, Germany; b Department of Neurosurgery, Martin-Luther-University Halle-Wittenberg, Ernst-Grube-Str. 40, 06097, Halle, Germany; c Department of Neurosurgery, Heidelberg University Hospital, Medical Faculty Heidelberg, Heidelberg University, Heidelberg, Germany

**Keywords:** Vestibular schwannoma, PANQOL, Health-related quality of life, Observation, Radiotherapy, Microsurgery, Patient-reported outcomes, Treatment outcomes

## Abstract

**Introduction:**

Vestibular schwannomas (VS) may cause hearing loss, balance disturbances, facial nerve dysfunction, and other symptoms that substantially affect health-related quality of life (HRQoL). As multiple management strategies exist, understanding treatment-specific HRQoL outcomes is essential for patient-centered decision-making.

**Research question:**

How do treatment options differ with respect to patient-reported HRQoL outcomes in vestibular schwannoma?

**Material and methods:**

A systematic search identified cross-sectional studies assessing HRQoL using the Penn Acoustic Neuroma Quality of Life (PANQOL) questionnaire. Eleven studies including 5156 patients met inclusion criteria. Random-effects meta-analyses were conducted for total PANQOL scores and key subdomains. Standardized mean differences (Hedges’ g) were calculated for pairwise treatment comparisons. Risk of bias was assessed using the Joanna Briggs Institute checklist.

**Results:**

Microsurgery was associated with a non-significant reduction in total PANQOL scores compared with radiotherapy (g = −0.43, p = 0.09) and a significant reduction compared with observation (g = −1.58, p = 0.01). No significant difference was observed between radiotherapy and observation (g = −0.34, p = 0.26). Subdomain analyses showed that microsurgery was negatively associated with facial function, balance, hearing, and pain, while anxiety, energy, and general health did not differ significantly between treatment options. Heterogeneity across analyses was substantial (I^2^ = 0.90–0.98). Overall study quality was moderate.

**Discussion and conclusion:**

For small to medium-sized VS, radiotherapy and observation were generally associated with better HRQoL preservation than microsurgery, particularly in facial function and pain domains. Surgery remains necessary for large or symptomatic tumors. HRQoL outcomes should be integrated into shared decision-making to inform patients about domain-specific treatment trade-offs.

## Highlights

• Microsurgical treatment is associated with lower HRQoL, with the greatest deficits observed in facial function and pain.
• Radiotherapy and observation show comparable HRQoL outcomes, particularly in small-to medium-sized tumors.
• Vestibular symptoms, hearing, facial nerve function, and anxiety are key determinants of HRQoL, independent of treatment.
• Taking the full patient burden into account, broader patient-centered outcomes should be used for shared decision-making.

## 1 Introduction

Vestibular schwannomas (VS), which originate from the vestibulocochlear nerve, are benign tumors that can lead to a range of neurological and functional impairments. These include hearing loss, imbalance, tinnitus, and facial dysfunction. These impairments can markedly affect patients’ health-related quality of life (HRQoL), regardless of tumor growth or treatment strategy (Carlson et al., 2015; Gauden et al., 2011). There is increasing evidence to suggest that psychological factors play a central role in shaping HRQoL outcomes in VS patients. Symptoms such as chronic pain, tinnitus, anxiety, depression and maladaptive coping strategies may have a greater impact on perceived well-being than neurological deficits alone (Thomas et al., 2023, 2024a, 2024b). Although surgical removal may provide reassurance by offering the perception of a definitive cure, the emotional and psychological consequences of living with VS often have a more profound impact on overall well-being than the chosen treatment modality itself (Carlson et al., 2021).

Therefore, assessing HRQoL in patients with VS is crucial in addition to traditional clinical outcome measures. Conventional evaluations primarily address objective parameters such as tumor control, hearing preservation, and facial nerve integrity (Gauden et al., 2011; Soulier et al., 2017; Pruijn et al., 2023). However, these metrics may not fully capture the broader effects of the disease on patients' daily functioning and psychosocial health. Recent studies have demonstrated that postoperative symptoms such as persistent headache and tinnitus are strongly associated with reduced perceived health benefits and increased psychological distress, independent of hearing loss or facial nerve function. In particular, postoperative headaches following retrosigmoid microsurgery have been shown to significantly reduce perceived health benefits, with pain-related helplessness, anxiety, depression, and personality traits such as low emotional stability contributing substantially to poorer outcomes (Thomas et al., 2023, 2024a). Similarly, postoperative tinnitus has been linked to increased emotional distress, somatisation tendencies, and reduced quality of life, underscoring the complex interaction between physical symptoms and psychological vulnerability (Thomas et al., 2024b).

Consequently, the use of validated, disease-specific instruments is essential. These instruments must accurately reflect the multifaceted burden experienced by this patient population, including emotional, cognitive, and pain-related dimensions. The Penn Acoustic Neuroma Quality of Life Questionnaire (PANQOL), introduced in 2010 (Shaffer et al., 2010), addresses this need by providing a disease-specific measure of HRQoL for individuals with VS. It has since been translated into multiple languages (Apa et al., 2023; Glaas et al., 2018; Medina et al., 2017; Pattankar et al., 2022). The PANQOL entails 26 items and evaluates several domains, including hearing, balance, facial function, and emotional and general health. However, some psychometric limitations have been reported. For instance, the "General Health" domain demonstrated low reliability, and the "Pain" domain, consisting of a single item, may not sufficiently capture the complexity of pain-related experiences. Additionally, strong inter-domain correlations have raised questions about the distinctiveness of certain constructs. Since the original validation primarily involved patients with small tumors, the findings may not be fully applicable to individuals with larger lesions (Carlson et al., 2022).

Given the increasing use of the PANQOL to evaluate disease-specific quality of life, a systematic synthesis of existing research is necessary to understand how HRQoL outcomes vary among patients receiving different treatments for VS. The objective of this systematic review is to identify and summarize cross-sectional studies that have employed the PANQOL to evaluate HRQoL in VS patients. Furthermore, when sufficient and comparable data were available, a meta-analysis was performed to quantitatively synthesize PANQOL domain scores across treatment modalities. The primary objective of this study is to compare PANQOL-reported outcomes between different treatment strategies and to explore factors, particularly psychological and symptom-related variables, that may contribute to observed differences in HRQoL.

## 2 Methods

### 2.1 Protocol and guidelines

The methodology followed the PRISMA guidelines and the Cochrane Handbook for Systematic Reviews of Interventions, see Supplementary Material 1 (Page et al., 2021). The protocol was registered with the Open Science Framework (Führes and Rutenkröger, 2024).

### 2.2 Information sources and search strategy

A comprehensive search of Web of Science, PSYNDEX, PsycINFO, MEDLINE, and the Cochrane Library was conducted to identify studies addressing disease-specific HRQoL in patients with VS. The search strategy combined controlled vocabulary (MeSH terms) and free-text terms related to *vestibular schwannoma* or *acoustic neuroma* and *quality of life*, including the PANQOL instrument. Publications in German or English up to 2025 were considered. The initial search was performed in November 2024 and updated in August 2025. Full search strategies for all databases are provided in Supplementary Material 2.

### 2.3 Eligibility criteria

Studies were eligible if they met the following criteria: (1) the study population included adults (aged ≥18 years) diagnosed with sporadic VS; (2) studies included patients who underwent surgery and/or received radiotherapy or wait-and-scan treatment; (3) disease-specific QoL was assessed using the PANQOL instrument; and (4) the study design used a cross-sectional or retrospective approach. Studies were excluded if they fell into the following categories: letters, editorials, protocols, case reports, or systematic reviews. Studies focusing on patients with neurofibromatosis 2 and those using non-specific QoL measures were excluded. We further excluded longitudinal studies due to methodological differences, potential biases, data inconsistencies, and concerns about study quality and relevance.

### 2.4 Study selection

Identified studies were screened and extracted using Rayyan. Three investigators (MJ, LB, LH) performed the initial assessment, with a third author (MR) providing oversight. Titles, abstracts and full-text articles were assessed independently, and discrepancies were resolved by consensus. Data extraction was performed independently by two reviewers (LB and LH), with disagreements resolved by a third author (MR).

### 2.5 Data synthesis

Data were extracted for all predefined outcomes related to HRQoL, including total PANQOL scores and domain-specific scores (hearing, balance, facial function, energy, and general health). For each outcome domain, all results reported in the included studies—across all measures, time points, and analyses—were sought whenever available. When multiple measures or time points were reported, results were extracted according to the predefined hierarchy prioritizing validated instruments and longest follow-up. Additional variables collected included participant characteristics (age, sex), tumor characteristics (size, laterality), treatment modality (observation, radiotherapy, microsurgery, combination therapies), follow-up duration, and funding sources. In cases where information was missing or unclear, assumptions were made based on available study details (e.g., mean age imputed from reported ranges or demographic tables). Data synthesis was carried out by MR and HF.

### 2.6 Quality assessment

The methodological quality of included studies was assessed by MR using the Joanna Briggs Institute (JBI) Critical Appraisal Checklist for Analytical Cross-Sectional Studies. This instrument is specifically designed to evaluate the internal validity of cross-sectional research and consists of eight items addressing key domains, including clarity of inclusion criteria, adequacy of participant and setting descriptions, validity and reliability of exposure and outcome measurements, identification and management of confounding factors, and appropriateness of statistical analyses. Each study was evaluated independently across all domains, allowing for an overall judgment of methodological quality.

### 2.7 Meta-analysis

A meta-analysis was conducted to compare PANQOL outcomes across the three management strategies. The primary outcome was the PANQOL total score, with secondary analyses conducted for individual PANQOL domains (facial function, balance, hearing, anxiety, energy, pain, and general health). Studies were deemed eligible for each synthesis based on predefined inclusion criteria and intervention characteristics, which were tabulated and compared against the planned groups. When multiple measures or time points were reported, results were extracted according to a predefined hierarchy prioritizing validated instruments and longest follow-up. Effect sizes were calculated using Hedges' g, accounting for standard errors. For studies reporting 95% confidence intervals rather than standard deviations, SDs were derived using standard statistical formulas. Random-effects models were employed to account for anticipated between-study heterogeneity. Heterogeneity was assessed using Tau^2^, I^2^, H^2^, and Cochran's Q statistics, with significance determined at a two-sided alpha level of 0.05. Data preparation included handling missing summary statistics and converting reported values to a common metric where required. Tabular summaries and visual displays (forest plots) were used to present individual study results and synthesized estimates. Potential sources of heterogeneity were explored through subgroup analyses based on treatment modality, follow-up duration, and tumor size. Sensitivity analyses were performed to assess the robustness of the synthesized results by excluding studies with high risk of bias or using alternative effect size calculations. All analyses were conducted using the meta-analysis module in SPSS Version 31.

## 3 Results

### 3.1 Study selection

**Fig. 1:**
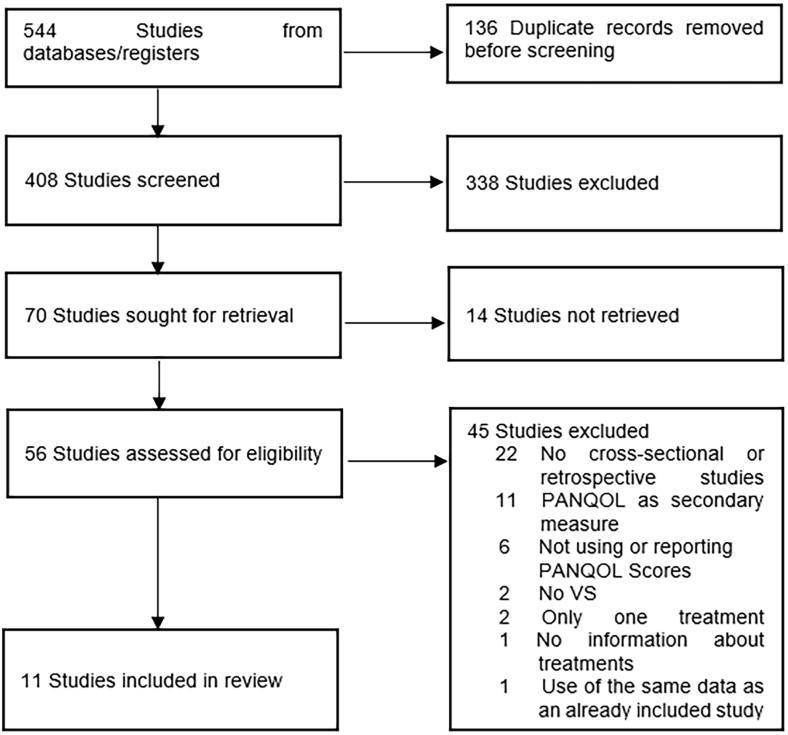
Flow diagram of studies identified, screened and included for this review.

Our systematic search yielded 544 references, of which 136 were identified as duplicates. Subsequent screening of titles and abstracts resulted in the exclusion of 338 articles as they did not meet the inclusion criteria. 70 studies were sought for retrieval, resulting in the exclusion of 14 studies. The remaining 56 articles were screened for full text, resulting in the exclusion of 45 articles (see Fig. 1). A total of 11 studies were eligible for inclusion in this review.

### 3.2 Study characteristics

A total of 5156 patients were analyzed across all included studies. Of those patients, 2153 underwent microsurgery, 907 received radiotherapy, and 1579 were managed with observation or interval scanning. Additionally, 96 patients received a combination of surgery and radiotherapy, 408 were newly diagnosed and had not received treatment by the time of assessment, and 13 underwent salvage therapy.

An overview of study characteristics is given in Table 1. Included studies encompassed diverse international cohorts, primarily from the United States (McLaughlin et al., 2013; Robinett et al., 2014; Carlson et al., 2018; Chweya et al., 2021) and Europe (Netherlands (Pruijn et al., 2021): France (Soulier et al., 2017):), United Kingdom (Ball et al., 2024; Lodder et al., 2018) and New Zealand (Nowacka et al., 2023). Sample sizes varied considerably, ranging from 50 participants (Nowacka et al., 2023) to >1000 patients (Chweya et al., 2021).

**Table 1:** Post-treatment patient characteristics, tumor features, and symptom burden across treatment modalities.

Source	Country	Surgical approach	Sample sizes (N) and age	Tumor sizes	Time since treatment (M, SD)	post-treatment				
						facial nerve function	hearing	dizziness	tinnitus	headaches
McLaughlin et al. (2013)	USA	/	186 (52% female, 98 OBS, 49 RT, 39 MS), Age: OBS 58 ± 13, RT 59 ± 12, MS 49 ± 14,	OBS: 8 ± 4.8 mm RT: 18 ± 5.9 mm MS: 22 ± 8.3 mm	Overall follow-up: 2.6	/	Audiometric data: Conservative: 35 dB (71%) Gamma knife: 53 dB (35%) Surgery: 50 dB (32%)	/	/	/
Robinett et al. (2014)	USA	Translabyrinthine (n = 70), Middle cranial fossa (n = 44), retrosigmoid (n = 43)	279 (48% female, 79 OBS, 43 RT, 157 MS), Age: OBS = 59.0 (range: 18-85), RT = 60.4 (range: 30-79), MS = 54.3 (21-84)	Mean tumor size was largest in MS group (p = 0.012)	Group 1 (0-5yrs), Group 2 (6-10yrs), Group 3 (>10yrs)	Facial weakness: MS 5.4%, OBS 3.9%, RT 4.8%, Facial pain, hypo/hyperesthesia: MS 13.6%, OBS 8.3%, RT 8.1%	Hearing loss: MS 96.4%, OBS 88.1&, RT 90.8%	Vertigo/imbalance: MS 55.3%, OBS 37.0%, RT 38.7%	Tinnitus: MS 49.6%, OBS 52.1%, RT 60.1%	/
Soulier et al. (2017)	Netherlands	/	807 (46% female, 469 OBS, 81 RT, 257 MS), Age (mean): OBS = 63.6, RT = 60.1, MS = 57.0	OBS: 5.9 mm RT: 10.3 mm MS: 17.6 mm	/	/	/	/	/	
Carlson et al. (2018)	USA	/	1284 (66% female, 303 OBS, 185 RT, 507 MS, 64 RT + MS 229 recently diagnosed), Age (mean) = 56.8	0–0.9 cm: 298 (26%) 1–1.9 cm: 375 (32%) 2–2.9 cm: 264 (23%) 3–3.9 cm: 130 (11%) >4 cm: 91 (8%)	0–5 yrs: 924 (72%) 6–10 yrs: 155 (12%) >11 yrs: 209 (16%)	/	/	/	/	/
Lodder et al. (2018)	UK, Netherlands	/	397 (female: 45.1%, 63 OBS, 94 RT, 185 MS, 17 RT + MS), Age (%): largest group: 61-70 years (38.2%)	n.a.	Overall M = 7 years, <6 years: 198; 6-10 years: 68; >10 years: 93	/	/	/	/	/
Pruijn et al. (2021)	Netherlands	/	174 (50% females, 130 OBS, 29 RT, 15 MS), 58.9 years	Koos 1: 52.3% Koos 2: 23.8% Koos 3: 13.1% Koos 4: 10.8%	1.1 ± 0.6 yrs (13 ± 6.8 months)	/	/	/	/	/
Chweya et al. (2021)	USA	/	1362 (64% female, 290 OBS, 271 RT, 686 MS, 115 recently diagnosed), Age (mean) = 59	Total tumor size (n = 1237): 0–0.9 cm 27%, 1–1.9 cm 35%, 2–2.9 cm 22%, 3–3.9 cm 9%, >4 cm 6% MS: 0–0.9 cm 14%, 1–1.9 cm 30%, 2–2.9 cm 29%, 3–3.9 cm 15%, >4 cm 11% RT: 0–0.9 cm 27%, 1–1.9 cm 51%, 2–2.9 cm 19%, 3–3.9 cm 2%, >4 cm 0% OBS: 0–0.9 cm 60%, 1–1.9 cm 30%, 2–2.9 cm 9%, 3–3.9 cm 1%, >4 cm 1% RD: 0–0.9 cm 20%, 1–1.9 cm 39%, 2–2.9 cm 20%, 3–3.9 cm 10%, >4 cm 0%	0–5 yrs: 903 (66%) 6–10 yrs: 229 (17%) >11 yrs: 230 (17%)	/	/	/	/	/
Fuentealba-Bassaletti et al. (2023)	Nether-lands	/	304 (55.4% female, 127 OBS, 42 RT, 122 MS, 13 Salvage Therapy), Age (mean) = 67.7	Intrameatal: 113 (36.7%) Small 0–10 mm: 63 (20.5%) Medium 11–20 mm: 72 (23.3%) Moderately large 21–30 mm: 32 (10.4%) Large + Giant >30 mm: 22 (7.1%) Missing: 7	Total cohort: 9 yrs OBS 10 yrs RT: 8 yrs MS: 10 yrs Salvage therapy: 9 yrs	/	/	DHI: Mild 37%, Moderate 18%, Severe 4%; 41% no vestibular symptoms	/	/
Nowacka et al. (2023)	New Zealand	/	52 (69.2% female, 13 OBS, 12 RT, 25 MS, 2 RT + MS), Age (mean) = 65.8	/	Time since last treatment: 12.4 (0.34)	Facial weakness 5.8% (3), numbness 5.8% (3), sharp facial pain 1.9% (1)	Hearing loss unilateral 42.3% (22), sudden hearing loss 28.8% (15)	Balance problems/dizziness: 25 (48.1%)	46.2% (24)	19.2% (10)
Ball et al. (2024)	UK	translabyrinthine (75 %), retrosigmoid (25 %)	134 (51.5% female, 86 OBS; 23 RT, 25 MS), Age (n) = <65: 58, ≥65: 69	Small–medium 1–3 cm; large >3 cm; categorized by ≤ 15 mm vs > 15 mm	Total follow-up: 4 yrs (IQR 2–5) OBS: 0–3 yrs n = 31, ≥4 yrs n = 45 RT, MS: 0–3 yrs n = 18, ≥4 yrs n = 28	PANQOL facial function: worse in larger tumors and MS (p = 0.039)	Hearing handicap: no significant differences between treatment modalities	Dizziness: female worse than male (p = 0.036); observation better than microsurgery/radiotherapy (p = 0.005)	Tinnitus: worse after RT (p = 0.018); <65 yrs worse scores	/

*Abbreviations.* OBS = Observation, RT = Radiotherapy/Radiosurgery; MS = Microsurgery; dB = decibel; cm = centimeter, mm = millimeter.

Patient demographics were generally comparable between treatment groups. Across studies, mean patient age ranged from approximately 50 to 70 years, with a consistent slight predominance of female participants, typically accounting for up to 70% of the study populations. Several studies reported that patients undergoing microsurgery tended to be younger than those treated with radiotherapy or managed by observation, reflecting established clinical selection patterns (Soulier et al., 2017; McLaughlin et al., 2013; Robinett et al., 2014; Lodder et al., 2018).

### 3.3 Differences between treatment groups

A summary of the main results of the studies is given in Table 2. Tumor size differed systematically by treatment modality. Patients managed with observation generally presented with the smallest tumors, often limited to intrameatal barely extrameatal lesions (Koos grades I–II tumors)(Carlson et al., 2018; Chweya et al., 2021; Pruijn et al., 2021)–(Carlson et al., 2018; Chweya et al., 2021; Pruijn et al., 2021). In contrast, surgically treated patients more often harbored larger tumors, commonly exceeding 20 mm in diameter, whereas radiotherapy cohorts typically exhibited intermediate tumor sizes.

**Table 2:** Additional outcome measures, statistical methods, and main findings of the included PANQOL studies.

Source	Additional measures used	Statistical methods	Main results
McLaughlin et al. (2013)	/	ANOVA and Tukey post hoc multiple comparisons test	PANQOL hearing domain higher in observation group (62 ± 26) vs surgery (47 ± 25); general and total PANQOL scores similar across groups; gamma knife and surgery QoL scores similar.
Robinett et al. (2014)	/	ANOVA, *t*-test, correlation	PANQOL higher for stereotactic radiation vs microsurgery and observation at 0–5 years (P = 0.009); no significant differences after 5 years; within radiation group, PANQOL lower at 6–10 years vs 0–5 years (P = 0.013).
Soulier et al. (2017)	/	Unpaired t testing and analysis of variance	Mean PANQOL scores were lowest for hearing, balance, and energy. Total PANQOL and the domains of hearing, balance, facial function, and energy differed significantly by treatment, with higher scores after observation compared to radiotherapy and microsurgery, particularly for small tumors. Self-reported symptoms, especially balance problems and vertigo, were strongly negatively correlated with overall HRQoL. No clear relationship between time since treatment and total PANQOL was observed.
Carlson et al. (2015)	SF-36	Linear regression models, mulitvariate models using stepwise selection with the P-value for a feature to enter or leave the models set to 0.05	Ongoing dizziness and headache strongly associated with lower PANQOL and SF-36 scores; treatment modality and sex did not significantly influence QoL.
Lodder et al. (2018)	/	Calculation of a composite score, Shapiro–Wilk normality test, unpaired *t*-test, ANOVA, *t*-test	Total cQOL did not differ by treatment (MS 58, RT 56, OBS 54, COMBO 49; p = 0.532); sub-domains facial function and balance differed significantly, with better scores after RT and OBS compared to MS; other domains were not significantly different. Long-term follow-up (>10 years) showed higher facial and energy scores.
Carlson et al. (2018)	/	Two-sample t, Wilcoxon rank sum, x^2^-test, ANOVA, ANCOVA,Tukey–Kramer method	Observation cohort had highest PANQOL total score (65), multimodality therapy lowest (56), surgery (60), and radiosurgery (61) intermediate; no significant differences between single-modality therapies after adjusting for covariates.
Pruijn et al. (2021)	SF-36	Multiple linear regression analysis with the PANQOL domain scores as predictors for both the SF-36 PCS and MCS scores, Student's t- tests, chi-squared test	PANQOL total 59.6 ± 17.8; lowest scores in hearing (34.9), balance (55.9), general (56.0); strongest predictors of poor physical health: lack of energy, lower general health, headache, anxiety, balance; mental health most affected by anxiety, energy, facial nerve dysfunction, balance, headache.
Chweya et al. (2021)	/	Analysis of variance, X^2^, Kruskal-Wallis tests, analysis of covariance, Tukey-Kramer method, Dunnett method, linear model diagnostics	Total PANQOL differed by treatment (p = 0.024): microsurgery (60), radiosurgery (63), observation (65), RD (62); subdomains (facial function, balance, hearing loss, pain) differed significantly; non-tumor controls had higher PANQOL scores across multiple domains.
Fuentealba-Bassaletti et al. (2023)	DHI	X^2^-test with Bonferroni correction for multiple testing, Wilcoxon rank test, linear regression	59% experienced dizziness; DHI total negatively associated with PANQOL (0.7 point reduction per DHI point); DHI emotional subdomain strongest determinant of lower QoL; treatment modality had no clinically relevant effect on dizziness-related QoL.
Nowacka et al. (2023)	HADS-S	Two between-group ANOVAs; Post-hoc analyses (Bonferroni correction); contingency tables using Freeman-Halton Fischer test; Spearman's bivariate correlations; hierarchical regression model	PANQOL total 58.35 ± 20.96; highest scores: radiation, then observation, lowest surgery; significant difference in total PANQOL by treatment (F = 6.96, p = 0.002); facial dysfunction and anxiety scored highest; hearing loss and balance lowest.
Ball et al. (2024)	DHI, HHI, THI	Descriptive statistics, non-parametric tests, Wilcoxon–Mann–Whitney, Kruskal–Wallis tests, Fisher's Exact Test	Observation group had higher PANQOL (p = 0.001) and lower pain (p = 0.005); microsurgery and radiotherapy groups had worse balance; facial function worse with larger tumors (>15 mm, p = 0.039); hearing better in observation (median 56) vs radiotherapy (31) and surgery (38); anxiety and energy not affected by treatment.

*Abbreviations*. SF-36 = Short Form-36, DHI = Dizziness Handicap Inventory, HADS-D = Hospital Anxiety and Depression Scale, HHI = Headache Handicap Inventory, THI = Tinnitus Handicap Inventory.

The interval between treatment and HRQoL assessment varied widely among studies, ranging from less than one year to over a decade. Several cohorts reported median or mean follow-up durations between two and ten years, with longer follow-up periods more frequently observed in surgically treated patients (Robinett et al., 2014; Lodder et al., 2018; Fuentealba-Bassaletti et al., 2023).

Most studies relied primarily on the PANQOL as the disease-specific measure of HRQoL. Only a limited number of investigations incorporated additional patient-reported outcome measures, including the Short Form-36 (SF-36) (Pruijn et al., 2021), the Dizziness Handicap Inventory (DHI) (Ball et al., 2024; Fuentealba-Bassaletti et al., 2023), the Hearing Handicap Inventory (HHI) and Tinnitus Handicap Inventory (THI) (Ball et al., 2024), or the Hospital Anxiety and Depression Scale (HADS) (Nowacka et al., 2023).

Several studies have reported significant differences in HRQoL and symptom-specific outcomes between treatment modalities, especially during the early post-treatment period. Overall, HRQoL was lower after microsurgery than after radiotherapy or observation. Radiotherapy was often linked to more favorable short-to mid-term outcomes. Early differences between management strategies were most evident within the first five years after treatment; however, long-term differences appeared to attenuate over time (Robinett et al., 2014; Nowacka et al., 2023).

Treatment-related differences were most consistently observed at the subdomain level across studies. Facial function and balance-related quality of life were repeatedly reported as poorer following microsurgery than following radiotherapy or observation (Chweya et al., 2021; Ball et al., 2024; Lodder et al., 2018). Hearing-related quality of life also differed by management strategy in several cohorts. Observation generally yielded more favorable outcomes than active treatment, although the absolute differences were often modest. Pain-related impairments were more frequently reported after microsurgical treatment. Anxiety and energy domains, however, showed more variable patterns across studies.

Tumor characteristics and symptom burden emerged as important determinants of HRQoL, independent of treatment modality. Larger tumor size was consistently associated with poorer outcomes across multiple PANQOL domains, especially facial function and balance (Soulier et al., 2017; Lodder et al., 2018). Vestibular symptoms played a central role in shaping perceived QoL. Persistent dizziness was strongly associated with lower overall PANQOL scores and was identified as one of the most influential drivers of reduced HRQoL, particularly in the emotional and balance-related domains (Fuentealba-Bassaletti et al., 2023). Hearing-related quality of life was less consistently related to the treatment modality itself. Tinnitus severity was reported to be greater after radiotherapy and among younger patients in one cohort (Ball et al., 2024).

Psychological factors also substantially contribute to HRQoL outcomes. Patients newly diagnosed with the condition reported elevated anxiety levels and lower quality of life, regardless of treatment status. This indicates that the psychological impact of the initial diagnosis may substantially influence early HRQoL assessments (Carlson et al., 2018).

However, several studies reported limited or no clinically meaningful differences in overall HRQoL between treatment strategies. In some cohorts, treatment modality was not a significant predictor of total PANQOL scores once symptom burden was considered (Carlson et al., 2015). These findings suggest that differences in HRQoL are driven more by treatment-related symptoms, tumor characteristics, and psychological factors than by the treatment label itself.

### 3.4 Risk of bias

Overall, the methodological quality of the included cross-sectional studies was moderate (see Table 3). Most studies clearly defined their inclusion criteria and study populations, and they used valid and reliable instruments, primarily the PANQOL, to assess health-related quality of life. Outcome measurement was deemed appropriate in all included studies. However, several methodological limitations were identified. Confounding factors such as tumor size, time since treatment, and baseline symptom burden were inconsistently identified and variably addressed in the analyses. While larger studies frequently applied multivariable or adjusted analyses, smaller cohorts often relied on unadjusted comparisons. Additionally, most studies did not report strategies for managing missing data. The statistical analyses were generally appropriate for the study design; however, heterogeneity in reporting and subgroup definitions limited comparability across studies. No study was excluded due to a high risk of bias; however, these limitations should be considered when interpreting the pooled results.

**Table 3:** Risk of bias assessment of included studies using the JBI Risk of Bias tool.

Study	Clear inclusion criteria	Study setting & participants described	Exposure (treatment group) measured validly	Outcome (PANQOL) measured reliably	Confounders identified	Confounders addressed	Appropriate statistical analysis	Overall risk of bias
McLaughlin et al. (2013)	Yes	Yes	Yes	Yes	Yes	No	Yes	Moderate
Robinett et al., 2014	Yes	Yes	Yes	Yes	Yes	Partially	Yes	Moderate
Soulier et al., 2017	Yes	Yes	Yes	Yes	Yes	Partially	Yes	Moderate
Carlson et al., 2015	Yes	Yes	Yes	Yes	Yes	Yes	Yes	Low–Moderate
Carlson et al., 2018	Yes	Yes	Yes	Yes	Yes	Yes	Yes	Low
Pruijn et al., 2021	Yes	Yes	Yes	Yes	Yes	Yes	Yes	Low
Chweya et al., 2021	Yes	Yes	Yes	Yes	Yes	Yes	Yes	Low
Fuentealba-Bassaletti et al., 2023	Yes	Yes	Yes	Yes	Yes	Yes	Yes	Low
Nowacka et al., 2023	Yes	Yes	Yes	Yes	Yes	Partially	Yes	Moderate
Ball et al., 2024	Yes	Yes	Yes	Yes	Yes	Partially	Yes	Moderate

### 3.5 Meta-analysis

Six studies were included in pairwise meta-analytic comparisons, which were performed for the total PANQOL score and all domains. For the total PANQOL score, surgery was associated with significantly lower HRQoL compared to observation (g = −1.58, SE = 0.62, p = 0.01, 95% CI -2.80 to −0.36, Fig. 2A). The differences between surgery and radiotherapy (Fig. 2B), and between radiotherapy and observation (Fig. 2C), were not statistically significant.

**Fig. 2A:**
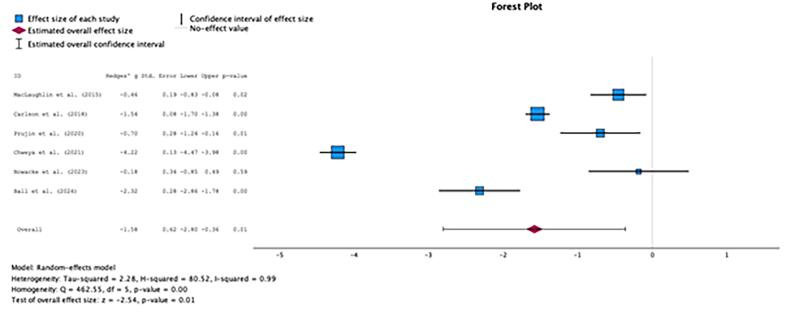
Comparison of total PANQOL scores between surgery and observation.

**Fig. 2B:**
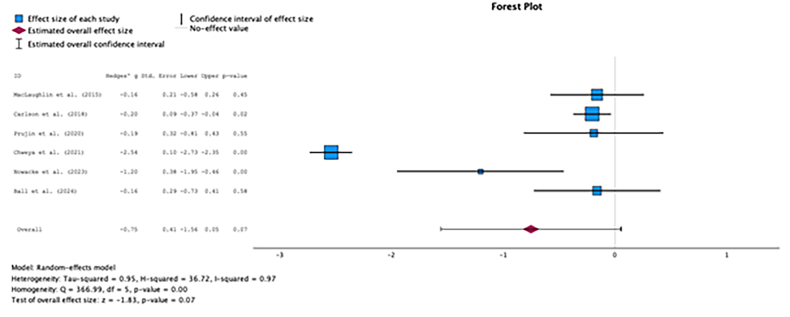
Comparison of total PANQOL scores between surgery and radiotherapy.

**Fig. 2C:**
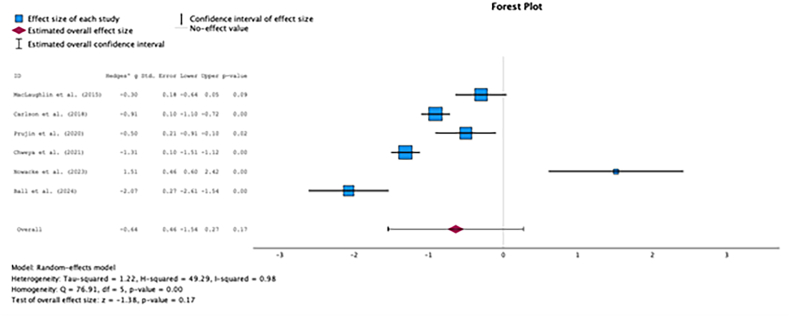
Comparison of total PANQOL scores between radiotherapy and observation.

Among the domain-specific analyses, significant differences were observed primarily for facial function and pain. Surgery was associated with significantly worse facial function compared to observation (g = −5.17, SE = 2.60, p = 0.047, 95% CI -10.27 to −0.08), while the comparison with radiotherapy showed a trend but did not reach significance (p = 0.061). Surgery also led to significantly worse pain scores compared to radiotherapy (g = −1.74, SE = 0.88, p = 0.048, 95% CI -3.47 to −0.01) and observation (g = −3.01, SE = 1.52, p = 0.047, 95% CI -5.98 to −0.04). All other comparisons for balance, hearing, anxiety, energy, and general health did not reach statistical significance in any pairwise analysis.

Overall, these results suggest that surgery is associated with lower HRQoL primarily in the facial function and pain domains, as well as in the overall PANQOL score, while radiotherapy and observation generally do not differ significantly. Heterogeneity was high across comparisons, with I^2^ values ranging from 0.90 to 0.98, suggesting substantial variability across studies.

## 4 Discussion

This systematic review included 5483 patients with VS across international cohorts, comparing outcomes after microsurgery, radiotherapy, or observation. The majority of patients were aged 50–70 years, with a slight female predominance. Surgical patients generally had larger tumors (Koos grade III - IV), while those under observation had smaller, often intrameatal or just extrameatal tumors (Koos grade I–II). Radiotherapy cohorts typically had intermediate tumor sizes. Follow-up times varied from under one year to more than a decade. This is supported by studies in higher-grade tumors, such as Foscolo et al. (2022), where patients with predominantly Koos III–IV VSs demonstrated clear associations between postoperative facial nerve dysfunction, vestibular impairment, and lower PANQOL domain scores, particularly in pain, facial function, and balance domains. These findings indicate that PANQOL captures clinically meaningful HRQoL impairment even in large tumor cohorts, although the pattern and magnitude of deficits may differ from those observed in small-to medium-sized tumors.

Overall HRQoL, as assessed by the PANQOL, varied by treatment modality. Meta-analytic comparisons revealed that surgery was associated with significantly lower PANQOL total scores compared with observation, with a trend toward lower scores versus radiotherapy. There was no statistically significant difference between radiotherapy and observation. Domain-specific analyses indicated that surgery particularly affected facial function and pain, with significant deficits compared with observation and radiotherapy. Other domains, including balance, anxiety, energy, general health, and hearing, showed no statistically significant differences between treatment groups. These findings support prior observations that surgery, particularly for larger tumors, is associated with greater impairment in key functional domains, whereas radiotherapy and observation tend to preserve HRQoL, especially in early post-treatment follow-up. Across comparisons, heterogeneity was substantial (I^2^ ranging from 0.90 to 0.98), reflecting variability in study design, sample characteristics, tumor size, and follow-up. Given the extremely high heterogeneity across studies, pooled effect estimates should be interpreted with considerable caution and are best regarded as exploratory and descriptive rather than precise quantitative summaries of treatment effects. This variability likely reflects differences in tumor size distribution, study design, follow-up duration, and unmeasured confounding.

Vestibular and auditory symptoms consistently emerged as major determinants of HRQoL. Dizziness and imbalance were strongly correlated with lower PANQOL scores, independent of treatment type (Ball et al., 2024; Fuentealba-Bassaletti et al., 2023). Hearing preservation and facial nerve function also remained key patient priorities, reinforcing the need to weigh functional outcomes alongside tumor control (Carlson et al., 2018; Pruijn et al., 2021). However, it is important to note that facial nerve dysfunction, particularly following microsurgical treatment, may undergo a partial recovery over time. This dynamic process may not be adequately captured by cross-sectional study designs. Consequently, the impact of facial function on HRQoL may be overestimated in studies assessing patients at early or heterogeneous postoperative time points (Franz et al., 2024; Lee et al., 2016). Similarly, variability in follow-up duration may be particularly relevant for pain-related outcomes and facial nerve dysfunction, as both may evolve over time due to postoperative recovery, adaptation or late sequelae. This temporal variability may have contributed to the differences observed across studies and may explain some of the treatment-related differences seen in these domains. Psychological factors, particularly anxiety related to the initial diagnosis, further influenced HRQoL irrespective of treatment (Robinett et al., 2014; Carlson et al., 2018).

Importantly, the cumulative impact of multiple concurrent symptoms may further reduce HRQoL, particularly in patients who have undergone surgery and often present with more advanced disease. Although the included studies primarily evaluated symptom domains individually, current analyses may not fully capture the additive or synergistic effects of coexisting symptoms. These findings are supported by qualitative evidence from patients who have undergone surgery for VS, which shows that physical, psychological and social challenges occur and interact with each other simultaneously. For example, emotional distress can lead to social isolation, while physical symptoms can increase psychological distress. This suggests that, in clinical reality, symptom burden is inherently multidimensional and may not be fully captured by domain-specific quantitative measures (Nieke et al., 2026).

Despite these insights, nearly all included studies relied exclusively on the PANQOL, which, while valuable for assessing symptom burden, has limitations. Moreover, as shown in Table 2, most studies only captured a limited range of symptoms beyond the PANQOL, rarely incorporating additional patient-reported outcome measures. These studies do not capture patient satisfaction, the alignment of treatment with personal goals, or subjective experiences during shared decision-making. It does not capture patient satisfaction, alignment of treatment with personal goals, or subjective experiences during shared decision-making. This limitation is further supported by qualitative evidence indicating that postoperative outcomes encompass more than just symptom-specific domains, incorporating complex interactions between physical, psychological, and social factors. Patients report long-term challenges such as anxiety, cognitive impairment, social withdrawal and changes to their work and relationships. These challenges are not captured by current PROMs, such as the PANQOL. Qualitative research further demonstrates that such patient-centered dimensions, including coping strategies, trust in clinicians, and expectations regarding functional preservation, significantly shape perceived HRQoL (Pruijn et al., 2023; Neve et al., 2021; Bras et al., 2025; Graham et al., 2018). This gap has motivated the development of newer instruments, such as the VSQOL, which explicitly measure satisfaction with care, treatment goal alignment, and the broader psychosocial impact of VS management (Carlson et al., 2022). Symptom-focused measures like the PANQOL may therefore underestimate the burden of vestibular dysfunction, tinnitus, and psychosocial stress, even when domain scores suggest favorable outcomes, particularly for radiotherapy and observation cohorts (Nowacka et al., 2023; Ball et al., 2024; Fuentealba-Bassaletti et al., 2023).

### 4.1 Strengths and limitations

This systematic review has several notable strengths. This study offers the first comprehensive synthesis of cross-sectional studies evaluating HRQoL in patients with vestibular schwannomas using a validated, disease-specific instrument (PANQOL), thereby facilitating domain-specific comparisons across treatment modalities. The incorporation of over 4000 patients from a variety of international cohorts serves to enhance the generalizability of the findings. Moreover, integrating narrative synthesis with meta-analytic effect size estimates facilitates the acquisition of both qualitative and quantitative insights into the impact of surgery, radiotherapy, and observation on overall and domain-specific HRQoL.

However, it is imperative to acknowledge the limitations that are inherent to this methodology. The included studies were exclusively cross-sectional in design, a limitation that precludes the ability to make causal inferences or to assess longitudinal changes in HRQoL. While this design choice improves comparability across treatment modalities, it limits insight into longitudinal HRQoL trajectories, including early postoperative deterioration, long-term recovery, and delayed treatment-related effects. The lack of longitudinal data further limits assessment of time-dependent recovery trajectories, particularly for facial nerve function and pain. In particular, confounding by indication represents a major limitation, as patients undergoing surgery generally harbored larger and more symptomatic tumors. A considerable degree of heterogeneity was observed in the study design, sample size, tumor characteristics, follow-up duration, and reporting of PANQOL subdomains. This heterogeneity complicates direct comparisons and contributes to high I^2^ values in the meta-analyses. A number of studies have focused on specific patient subsets, such as patients with small tumors, those who have been newly diagnosed, or those who have undergone specific treatment modalities. However, these studies may reduce the external validity of the research. Additionally, while the PANQOL is designed to assess a wide range of symptoms, including hearing, balance, facial nerve function, pain, and energy, it does not encompass all the elements necessary for a comprehensive evaluation of HRQoL. Specifically, it lacks the capacity to measure patient-centered outcomes such as treatment satisfaction, alignment with personal goals, and psychosocial well-being. As a result, its scope is limited in terms of fully evaluating the comprehensive nature of HRQoL. The PANQOL has several known psychometric limitations that must be considered when interpreting results. These include the limited reliability of the general health domain, the fact that pain is assessed using a single item, and the primary validation of the tool in cohorts that predominantly consisted of small vestibular schwannomas. Consequently, these limitations may reduce the ability to detect subtle differences between groups and should be taken into account when drawing conclusions from PANQOL-based comparisons. However, PANQOL has also been applied in surgically treated cohorts with predominantly large and giant VS. In the study by Foscolo et al., 70% of patients presented with Koos III–IV tumors, and PANQOL domains showed strong correlations with the total score. Importantly, postoperative facial nerve dysfunction and vestibular impairment were associated with significantly lower scores in the facial dysfunction, pain, and balance domains. These findings suggest that PANQOL remains sensitive to clinically relevant postoperative morbidity even in higher-grade tumors, while also highlighting the importance of interpreting results in a tumor-size–stratified context.

### 4.2 Clinical implications

The findings support a treatment approach that is adapted to the size and risk of the tumor rather than one that is driven solely by the presence of symptoms. Although vestibular and auditory symptoms, such as dizziness or tinnitus, may occur in patients with small VSs, the decision to perform surgery is primarily based on complications related to tumor size, including cerebellar compression or hydrocephalus. For patients with small-to medium-sized tumors without mass effect, observation or radiotherapy is preferable, as these strategies are associated with better facial nerve function preservation and lower treatment-related pain. For larger tumors that cause neurological compromise, surgical intervention is necessary. In these cases, patients should be counseled about potential trade-offs in quality of life related to specific domains, such as facial nerve function, balance, and pain. According to current EANO recommendations (Goldbrunner et al., 2020), a reasonable strategy to balance tumor control with functional preservation is planned subtotal resection followed by adjuvant radiotherapy, although postoperative discomfort may still occur. These findings highlight the importance of incorporating HRQoL outcomes into shared decision-making to align treatment choices with patient priorities.

## 5 Conclusion

This systematic review and meta-analysis demonstrates that HRQoL in patients with VS differs significantly across treatment modalities. Microsurgical treatment, typically applied to larger tumors, is associated with greater impairment in overall HRQoL, particularly affecting facial function and pain, whereas radiotherapy and observation appear associated with comparatively preserved HRQoL in predominantly small-to medium-sized tumors, although this finding is based on cross-sectional evidence with heterogeneous follow-up and is subject to important limitations. In particular, late radiation-induced effects and long-term psychological burden associated with tumor surveillance under observation may not be adequately captured in the available evidence. Vestibular and auditory symptoms, facial nerve function, and psychological factors are key determinants of HRQoL irrespective of treatment choice. These findings underscore the importance of integrating validated patient-reported outcomes into shared decision-making and highlight the need for longitudinal and more comprehensive, patient-centered assessment tools to better capture the full impact of VS management.

## Ethics approval and consent to participate

Not applicable. This review is based exclusively on previously published studies and does not involve the collection of new data from human participants.

## Consent for publication

Not applicable.

## Availability of data and material

All data generated or analyzed during this study are included in this published article and its supplementary information files.

## Author contributions

MR**:** Conceptualization, Methodology, Data curation, Formal analysis, Investigation, Writing – original draft, Writing – review & editing.

LB: Literature search, Screening, Data curation, Writing – review & editing.

LH: Screening, Data curation, Writing – review & editing.

MS: Writing – original draft, Writing – review & editing.

## Funding

The authors received no specific funding for this work.

## Declaration of competing interest

The authors declare that they have no known competing financial interests or personal relationships that could have appeared to influence the work reported in this paper.

## List of abbreviations

VS: vestibular schwannoma
HRQoL: health-related quality of life
PANQOL: Penn Acousticus Neurinoma Quality of Life Questionnaire
VSQOL: Vestibular Schwannoma Quality of Life Index
EANO: European Association of Neuro-Oncology

## References

[bib1] Apa E., Maccarrone F., Gherpelli C., Sacchetto L., Monzani D., Palma S. (2023). Italian validation of the penn acoustic neuroma quality of life scale (PANQOL-It). Acta Otorhinolaryngol Ital Organo Uff Della Soc Ital Otorinolaringol E Chir Cerv-facc.

[bib2] Ball J.F., Low J.C.M., Kasbekar A.V., Lesser T.H. (2024). Comparison of quality of life in vestibular schwannoma patients managed with observation, radiotherapy or microsurgery. J. Laryngol. Otol..

[bib3] Bras I.J., Joosen M.C., Rutten G.-J.M., van Vugt I.J., Sitskoorn M.M., Boele F.W. (2025). A thematic analysis of shared decision-making in consultations with patients with a presumed brain tumor and neurosurgeons. Neuro-Oncol Pract. Oxford University Press US.

[bib4] Carlson M.L., Tveiten Ø.V., Driscoll C.L., Goplen F.K., Neff B.A., Pollock B.E. (2015). What drives quality of life in patients with sporadic vestibular schwannoma?. Laryngoscope.

[bib5] Carlson M.L., Tombers N.M., Kerezoudis P., Celda M.P., Lohse C.M., Link M.J. (2018). Quality of life within the first 6 months of vestibular schwannoma diagnosis with implications for patient counseling. Otol Neurotol Off Publ Am Otol Soc Am Neurotol Soc Eur Acad Otol Neurotol. United States.

[bib6] Carlson M.L., Barnes J.H., Nassiri A., Patel N.S., Tombers N.M., Lohse C.M. (2021). Prospective study of disease-specific quality-of-life in sporadic vestibular schwannoma comparing observation, radiosurgery, and microsurgery. Otol Neurotol. LWW.

[bib7] Carlson M.L., Lohse C.M., Link M.J., Tombers N.M., McCaslin D.L., Saoji A.A. (2022). Development and validation of a new disease-specific quality of life instrument for sporadic vestibular schwannoma: the Mayo Clinic Vestibular Schwannoma Quality of Life Index. J Neurosurg. American Association of Neurological Surgeons.

[bib8] Chweya C.M., Tombers N.M., Lohse C.M., Link M.J., Carlson M.L. (2021). Disease-specific quality of life in vestibular schwannoma: a national cross-sectional study comparing microsurgery, radiosurgery, and observation. Otolaryngol Neck Surg.

[bib9] Foscolo V., de Gennaro L., Murri A., Speranzon L., Signorelli F., Quaranta N. (2022). Postoperative impact of pontocerebellar angle surgery on the quality of life in patients with Vestibular Schwannoma. Audiol Res. Switzerland.

[bib10] Franz L., Montino S., Agostinelli A., Tealdo G., Cazzador D., Zanoletti E. (2024). Functional outcomes and self-reported quality of life in patients with facial nerve impairment following vestibular schwannoma surgery. Diagnostics.

[bib11] Fuentealba-Bassaletti C., Neve O.M., van Esch B.F., Jansen J.C., Koot R.W., van Benthem P.P.G. (2023). Vestibular complaints impact on the long-term quality of life of vestibular schwannoma patients. Otol Neurotol Off Publ Am Otol Soc Am Neurotol Soc Eur Acad Otol Neurotol. United States.

[bib12] Führes H., Rutenkröger M. (2024). Differential impacts of microsurgery and radiotherapy on disease-specific quality of life in vestibular schwannoma patients: a systematic review. OSF Registries.

[bib13] Gauden A., Weir P., Hawthorne G., Kaye A. (2011). Systematic review of quality of life in the management of vestibular schwannoma. J. Clin. Neurosci..

[bib14] Glaas M.F., Schäfer R., Jansen P., Franz M., Stenin I., Klenzner T. (2018). Quality of life after Translabyrinthine Vestibular Schwannoma resection-reliability of the German PANQOL questionnaire. Otol Neurotol Off Publ Am Otol Soc Am Neurotol Soc Eur Acad Otol Neurotol. United States.

[bib15] Goldbrunner R., Weller M., Regis J., Lund-Johansen M., Stavrinou P., Reuss D. (2020). EANO guideline on the diagnosis and treatment of vestibular schwannoma. Neuro Oncol..

[bib16] Graham M.E., Westerberg B.D., Lea J., Hong P., Walling S., Morris D.P. (2018). Shared decision making and decisional conflict in the management of vestibular schwannoma: a prospective cohort study. J. Otolaryngol. Head Neck Surg..

[bib17] Lee S., Seol H.J., Park K., Lee J.-I., Nam D.-H., Kong D.-S. (2016). Functional outcome of the facial nerve after surgery for vestibular schwannoma: prediction of acceptable long-term facial nerve function based on immediate postoperative facial palsy. World Neurosurg..

[bib18] Lodder W.L., van der Laan B.F.A.M., Lesser T.H., Leong S.C. (2018). The impact of acoustic neuroma on long-term quality-of-life outcomes in the United Kingdom. Eur Arch Oto-Rhino-Laryngol Off J Eur Fed Oto-Rhino-Laryngol Soc EUFOS Affil Ger Soc Oto-Rhino-Laryngol - Head Neck Surg. Germany.

[bib19] McLaughlin E.J., Bigelow D.C., Lee M.Y., Ruckenstein M.J. (2013). Quality-of-Life differences by treatment groups for Acoustic neuroma patients. J. Neurol. Surg. Part B Skull Base.

[bib20] Medina M.D.M., Carrillo A., Polo R., Fernandez B., Alonso D., Vaca M. (2017). Validation of the Penn Acoustic Neuroma quality-of-life Scale (PANQOL) for spanish-speaking patients. Otolaryngol--Head Neck Surg Off J Am Acad Otolaryngol-Head Neck Surg. England.

[bib21] Neve O.M., Soulier G., Hendriksma M., Van Der Mey A.G.L., van Linge A., Van Benthem P.P.G. (2021). Patient-reported factors that influence the vestibular schwannoma treatment decision: a qualitative study. Eur. Arch. Otorhinolaryngol..

[bib22] Nieke N., Brandes L., Wandke S., Scholl I., Rutenkröger M. (2026). Understanding post-surgical recovery in Vestibular Schwannoma: a qualitative exploration of patient experiences. Brain Spine.

[bib23] Nowacka A., Barker-Collo S., Miles A., Ben-Harosh L. (2023). The effect of symptomatology and mental wellbeing on quality of life in people with acoustic neuroma. J Clin Neurosci Off J Neurosurg Soc Australas. Scotland.

[bib24] Page M.J., McKenzie J.E., Bossuyt P.M., Boutron I., Hoffmann T.C., Mulrow C.D. (2021). The PRISMA 2020 statement: an updated guideline for reporting systematic reviews. BMJ.

[bib25] Pattankar S., Churi O., Misra B.K. (2022).

[bib26] Pruijn I.M., Kievit W., Hentschel M.A., Mulder J.J., Kunst H.P. (2021). What determines quality of life in patients with vestibular schwannoma?. Clin. Otolaryngol..

[bib27] Pruijn I.M.J., van Heemskerken P., Kunst H.P.M., Tummers M., Kievit W. (2023). Patient-preferred outcomes in patients with vestibular schwannoma: a qualitative content analysis of symptoms, side effects and their impact on health-related quality of life. Qual Life Res Int J Qual Life Asp Treat Care Rehabil. Netherlands.

[bib28] Robinett Z.N., Walz P.C., Miles-Markley B., Moberly A.C., Welling D.B. (2014). Comparison of long-term quality-of-life outcomes in vestibular schwannoma patients. Otolaryngol--Head Neck Surg.

[bib29] Shaffer B.T., Cohen M.S., Bigelow D.C., Ruckenstein M.J. (2010). Validation of a disease‐specific quality‐of‐life instrument for acoustic neuroma: the Penn Acoustic Neuroma quality-of-life Scale. Laryngoscope.

[bib30] Soulier G., van Leeuwen B.M., Putter H., Jansen J.C., Malessy M.J.A., van Benthem P.P.G. (2017). Quality of life in 807 patients with vestibular schwannoma: comparing treatment modalities. Otolaryngol--Head Neck Surg Off J Am Acad Otolaryngol-Head Neck Surg. England.

[bib31] Thomas M., Rampp S., Scheer M., Strauss C., Prell J., Schönfeld R. (2023). Premorbid psychological factors associated with long-term postoperative headache after microsurgery in vestibular Schwannoma—A retrospective pilot Study. Brain Sci..

[bib32] Thomas M., Führes H., Scheer M., Rampp S., Strauss C., Schönfeld R. (2024). Perceived health benefits in vestibular schwannoma patients with long-term postoperative headache: insights from personality traits and pain Coping—A cross-sectional Study. J Pers Med.

[bib33] Thomas M., Scheer M., Rampp S., Strauss C., Schönfeld R., Leplow B. (2024). Psychological factors and long-term tinnitus handicap in vestibular schwannoma patients after retrosigmoid microsurgery - a cross-sectional study. Int J Audiol. England.

