# Supplementary material for: Health-related quality of life disparities among vestibular schwannoma patients under different treatment regimens: A systematic review and meta-analysis
Source: Brain Spine. 2026 May 3;6:106074. doi: 10.1016/j.bas.2026.106074 (PMC13157091; doi:10.1016/j.bas.2026.106074)
Supplement: Multimedia component 2 [file mmc2.docx]

Supplementary Material 2.

Full search strategies.

PubMed: ((vestibular schwannoma) OR (acoustic neuroma)) AND ((quality of life) OR (PANQOL)) ("neuroma, acoustic"[MeSH Terms] OR ("neuroma"[All Fields] AND "acoustic"[All Fields]) OR "acoustic neuroma"[All Fields] OR ("vestibular"[All Fields] AND "schwannoma"[All Fields]) OR "vestibular schwannoma"[All Fields] OR ("neuroma, acoustic"[MeSH Terms] OR ("neuroma"[All Fields] AND "acoustic"[All Fields]) OR "acoustic neuroma"[All Fields] OR ("acoustic"[All Fields] AND "neuroma"[All Fields]))) AND ("quality of life"[MeSH Terms] OR ("quality"[All Fields] AND "life"[All Fields]) OR "quality of life"[All Fields] OR "PANQOL"[All Fields])

Web of Science: vestibular schwannoma AND PANQOL AND quality of life

PSYNDEX: vestibular schwannoma AND quality of life

Cochrane: vestibular schwannoma AND quality of life
